# Rapid and Specific Enrichment of Culturable Gram Negative Bacteria Using Non-Lethal Copper-Free Click Chemistry Coupled with Magnetic Beads Separation

**DOI:** 10.1371/journal.pone.0127700

**Published:** 2015-06-10

**Authors:** Emilie Fugier, Audrey Dumont, Annie Malleron, Enora Poquet, Jordi Mas Pons, Aurélie Baron, Boris Vauzeilles, Sam Dukan

**Affiliations:** 1 Aix Marseille Université, Laboratoire de Chimie Bactérienne (UMR 7283), Institut de Microbiologie de la Méditerranée (IMM), CNRS, 31 Chemin Joseph Aiguier—13402, Marseille, France; 2 CNRS and Univ Paris-Sud, Laboratoire de Synthèse de Biomolécules, Institut de Chimie Moléculaire et des Matériaux d’Orsay, UMR 8182, 91405, Orsay, France; 3 Centre de Recherche de Gif, Institut de Chimie des Substances Naturelles du CNRS, Avenue de la Terrasse, 91198, Gif-sur-Yvette, France; Duke University Marine Laboratory, UNITED STATES

## Abstract

Currently, identification of pathogenic bacteria present at very low concentration requires a preliminary culture-based enrichment step. Many research efforts focus on the possibility to shorten this pre-enrichment step which is needed to reach the minimal number of cells that allows efficient identification. Rapid microbiological controls are a real public health issue and are required in food processing, water quality assessment or clinical pathology. Thus, the development of new methods for faster detection and isolation of pathogenic culturable bacteria is necessary. Here we describe a specific enrichment technique for culturable Gram negative bacteria, based on non-lethal click chemistry and the use of magnetic beads that allows fast detection and isolation. The assimilation and incorporation of an analog of Kdo, an essential component of lipopolysaccharides, possessing a bio-orthogonal azido function (Kdo-N_3_), allow functionalization of almost all Gram negative bacteria at the membrane level. Detection can be realized through strain-promoted azide-cyclooctyne cycloaddition, an example of click chemistry, which interestingly does not affect bacterial growth. Using *E*. *coli* as an example of Gram negative bacterium, we demonstrate the excellent specificity of the technique to detect culturable *E*. *coli* among bacterial mixtures also containing either dead *E*. *coli*, or live *B*. *subtilis * (as a model of microorganism not containing Kdo). Finally, in order to specifically isolate and concentrate culturable *E*. *coli* cells, we performed separation using magnetic beads in combination with click chemistry. This work highlights the efficiency of our technique to rapidly enrich and concentrate culturable Gram negative bacteria among other microorganisms that do not possess Kdo within their cell envelope.

## Introduction

The development of rapid and specific detection methods for pathogenic bacteria of interest, which in most cases are present at very low concentration, in complex samples, is currently a competitive and very intensive field of research. All the techniques currently used require a culture-based pre-enrichment step to reach, by multiplication, enough bacteria to allow detection and identification. Many efforts focus on the possibility to shorten this preliminary enrichment step.

Microbiological controls are for example required in food processing, water quality assessment or clinical pathology and need to be as fast as possible. Among the variety of strategies proposed to improve efficiency and rapidity of the process, the use of magnetic beads for sample purification and concentration seems to be promising. The extraction and concentration of a target organism from a sample by magnetic beads coated with specific monoclonal antibodies, named ImmunoMagnetic Separation (IMS), have been previously reported [1–4]. The specificity of the antibody coupled with the magnetic properties of the bead, allows a bacterium of interest to be separated from solid material or background microflora, and concentrated into a smaller sample volume. Combined with IMS, many detection methods can be used as a second step, to quantify and/or identify the captured bacteria. Most of the time, this approach has been directly applied in combination with selective culture-based methods [5], or the beads have been used as solid support for enzyme-linked immunosorbent assay (ELISA) [6, 7]. In addition, identification of captured bacteria can be realized by PCR after an elution step [8–13]. Other methods of identification involve a secondary antibody to form a sandwich complex for further use of detection tools such as ATP-bioluminescence [14] or electrochemiluminescence [15, 16]. These strategies have also been used with promising results to estimate the quantity of bacteria present in a sample.

Nevertheless, these approaches tend to misestimate the actual number of culturable bacteria present in a given sample, since IMS does not permit differentiation between culturable and non-culturable bacteria, which is a critical point for microbiological quality controls. All regulations in force only take into account the concentration of culturable bacteria of interest. In addition, it has been reported that direct immunomagnetic capture of specific bacteria is drastically decreased in the presence of other organisms which can be critical and problematic if the pathogenic bacteria of interest is weakly present in the sample. This can lead to a non capture of the pathogenic organism resulting in false negative analysis [17]. Therefore, the two major drawbacks displayed by IMS are interference in the presence of other organisms and no possible segregation between culturable, and non-culturable bacterial targets.

The concept of click chemistry, which was introduced at the turn of the century by Kolb, Finn and Sharpless [18], relies on the use of highly efficient exergonic reactions to connect molecular modules. Copper(I)-catalyzed Azide Alkyne Cycloaddition (CuAAC) [19, 20] very quickly emerged as the prototypic reaction of click chemistry, and found applications in numerous fields including bioconjugation and *in vivo* chemistry, since the azido and the alkyne groups could be considered as bioorthogonal. Indeed, these groups are inert in most biological environments, but still chemically reactive in specific conditions. The major limitation of CuAAC is copper itself, which shows cellular toxicity. This prompted the development of non-lethal, copper-free Strain-promoted Alkyne Azide Cycloaddition (SPAAC) [21]. This concept is based on the use of a strained alkyne, which is able to react spontaneously with organic azides, leading to the formation of a covalent linkage.

To our knowledge no technique allowing separation of culturable and non-culturable bacteria is currently available. An innovative method permitting to reach this objective could rely on surface functionalization of culturable bacteria in order to hook them onto magnetic beads using for example non-lethal click chemistry. Interestingly, we have recently demonstrated the possibility to functionalize the surface of almost all Gram negative bacteria by culturing them with 8-azido-3,8-dideoxy-d-manno-octulosonate (Kdo-N_3_), an analog of Kdo presenting a bio-orthogonal azido function (N_3_) [22]. The Kdo unit is a specific and essential component of the inner core of LPS [23, 24] of all Gram negative species excepted the *Shewanella* genus, which has recently been shown to use 8-amino-8-deoxy-Kdo instead [25, 26]. Moreover, among other organisms, presence of Kdo is extremely rare, except for algae and higher plants.

Based on these previous results, the present study associates click chemistry, bacteria and the use of magnetic beads to develop a new and fast method for concomitant collection and enrichment of culturable Gram negative bacteria present in a complex sample. For the purpose of this study, *E*. *coli* has been used as a model of Gram negative bacterium, and *B*. *subtilis* as a representative organism that does not contain Kdo.

## Materials and Methods

### Bacterial strains and growth conditions

The bacterial strains used in this study were derivatives from *E*. *coli* K12 (MG1655), Kan^r^ (kanamycin resistant), m-cherry Chl^r^ (chloramphenicol resistant) as well as GFP (green fluorescent protein) *B*. *subtilis* Chl^r^ which was kindly provided by Annick Guiseppi (LCB, IMM, Marseille, France). Bacteria were inoculated in LB medium with their corresponding antibiotics and grown in a rotary shaker (160 rpm) at 37°C for 5 h (over day cultures) or 16 h (overnight cultures). m-cherry dead *E*. *coli* were obtained after autoclaving at 121°C for 20 min.

### Copper-free click chemistry

#### Overnight Kdo-N_3_ incorporation

Over day cultures were diluted 100 times in fresh medium (final volume 200 μl) with or without addition of Kdo-N_3_ (1 mM) and incubated overnight at 37°C (S1 Fig). Depending on experiments, bacterial mixtures were prepared as follows: 10^6^ m-cherry dead Chl^r^ *E*. *coli* or 1x10^6^ to 3x10^6^ culturable GFP Chl^r^ *B*. *subtilis* together with 10^6^, 10^3^, 10^2^ or 10^1^ culturable Kan^r^ *E*. *coli*, respectively. Cultures (mixed or not) were washed 3 times with phosphate buffer (0.05 M, pH 7.4) by centrifugation at 12,000 rpm for 2 min at room temperature before incubation for 30 min at 37°C in 10 μl of a sulfo-azadibenzocyclooctyne-biotin conjugate (named in all the document, sulfo-DBCO-biotin; 1 mM, Jena Bioscience) (S1 Fig). Cells were finally washed 3 times with phosphate buffer by centrifugation at 12,000 rpm for 2 min at room temperature and prepared for microscopy analysis or incubated with streptavidin magnetic beads (see below).

#### 2 to 4 hours of Kdo-N_3_ incorporation

Overnight cultures of *E*. *coli* were diluted 100 times in fresh medium (final volume 10 ml) and grown at 37°C until OD_600nm_ reached 0.1. After serial dilutions, 5 cells were incubated with or without Kdo-N_3_ (0 mM, 10 mM, 25 mM or 50 mM) and bacterial growth was analyzed by CFU (colony forming unit) plate counting over a 6 h period to determine the maximal non-toxic Kdo-N_3_ concentration. Then, 5 cells were incubated with or without 25 mM Kdo-N_3_ (20 μl final volume) at 37°C for 2, 3 and 4 h. Regularly a 2 μl aliquot was subjected to copper-free click ligation with sulfo-DBCO-biotin as described above, followed by incubation with streptavidin magnetic beads (see below).

### Labeling with anti-biotin antibodies

Assimilation of Kdo-N_3_ was visualized by copper-free click chemistry using sulfo-DBCO-biotin followed by labeling with a mouse anti-biotin Alexa Fluor 488 or 594 antibody conjugate (Jackson ImmunoResearch) for 30 min at room temperature. Bacteria were also visualized by DAPI staining.

### Fluorescence microscopy

Bacteria were deposited onto glass microscope slides and then covered with a thin (1 mm thick) semisolid 1% agar pad made with dilute LB (1/10 in phosphate buffer (0.05 M, pH 7.4). Images were recorded with a motorized epifluorescence microscope (Nikon TE2000-E-PFS, Nikon, France) equipped with a CoolSNAP HQ 2 camera (Roper Scientific, Roper Scientific SARL, France) and a 100x/1.4 DLL (dark low low) objective. Excitation light was emitted by a 120 W metal halide light and signal was monitored using appropriate filters. Digital analysis and image processing were conducted by a custom automation script (Visual Basic) under Metamorph 7.5 (Molecular Devices, Molecular Devices France, France), as previously described [27].

### Isolation using streptavidin magnetic beads

Bacteria were incubated in the presence of 2x10^7^ dynabeads M-280 streptavidin (Invitrogen) (final volume 1 ml of phosphate buffer 0.05 M, pH 7.4 containing 0.01% BSA) for 1 h at room temperature on a rotary shaker (250 rpm). The supernatant was recovered by retention of the magnetic beads during 3 min of equilibration with a magnet. After two washes with phosphate buffer (0.05 M, pH 7.4, room temperature) containing BSA (0.01%), the magnetic beads were resuspended in phosphate buffer (1 ml). The presence of culturable *E*. *coli* or *B*. *subtilis* in the supernatant or on the magnetic beads was monitored both by enumeration of CFUs and observation by microscopy after anti-biotin antibody-Alexa Fluor 488 or 594 and DAPI labeling. Statistical unpaired T-test analyses were performed among values obtained in the supernatant and on the magnetic beads.

## Results

### Specific detection of culturable *E*. *coli* through azide-cyclooctyne ligation

**Fig 1:**
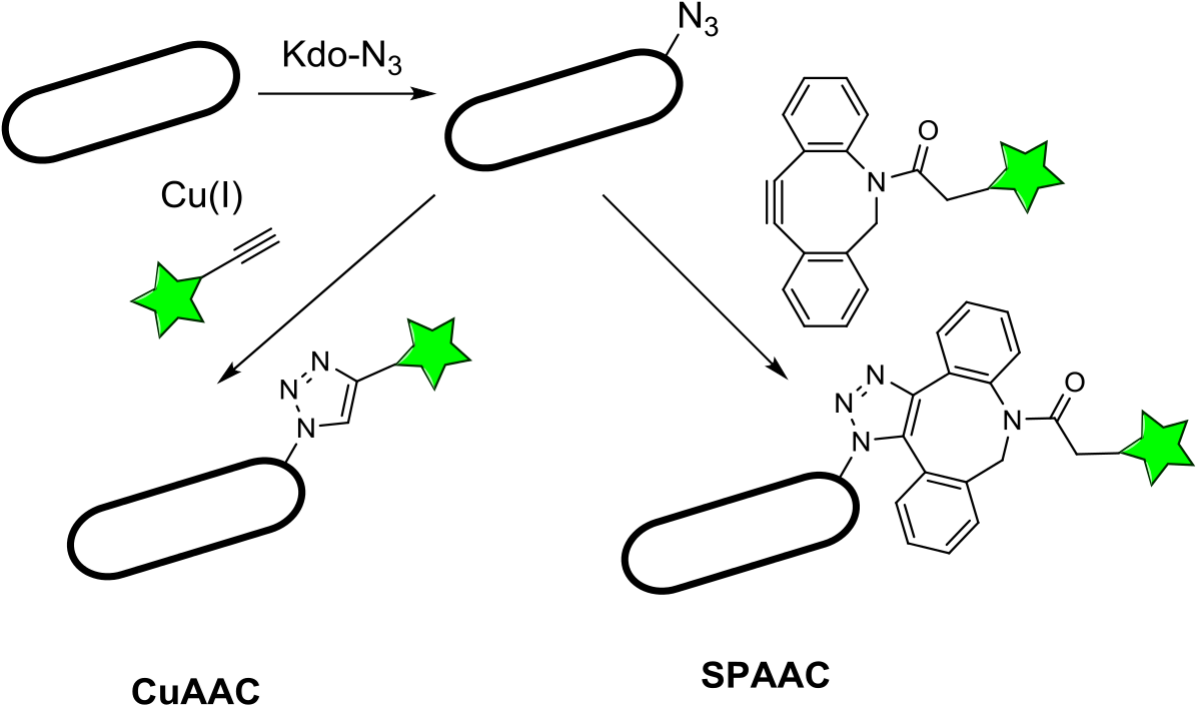
Schematic representation of metabolic lipopolysaccharide labeling using Kdo-N_3_. Culture of *E*. *coli* in the presence of Kdo-N_**3**_ results in incorporation of the bioorthogonal *azido* function with the bacterial LPS. This incorporation can be further visualized by Copper-catalyzed Azide Alkyne Cycloaddition (CuAAC), in the presence of Cu(I) and a terminal alkyne. Alternatively, Strain-Promoted Alkyne Azide Cycloaddition (SPAAC) with a cyclooctyne-type reagent results in copper-free ligation.

**Fig 2:**
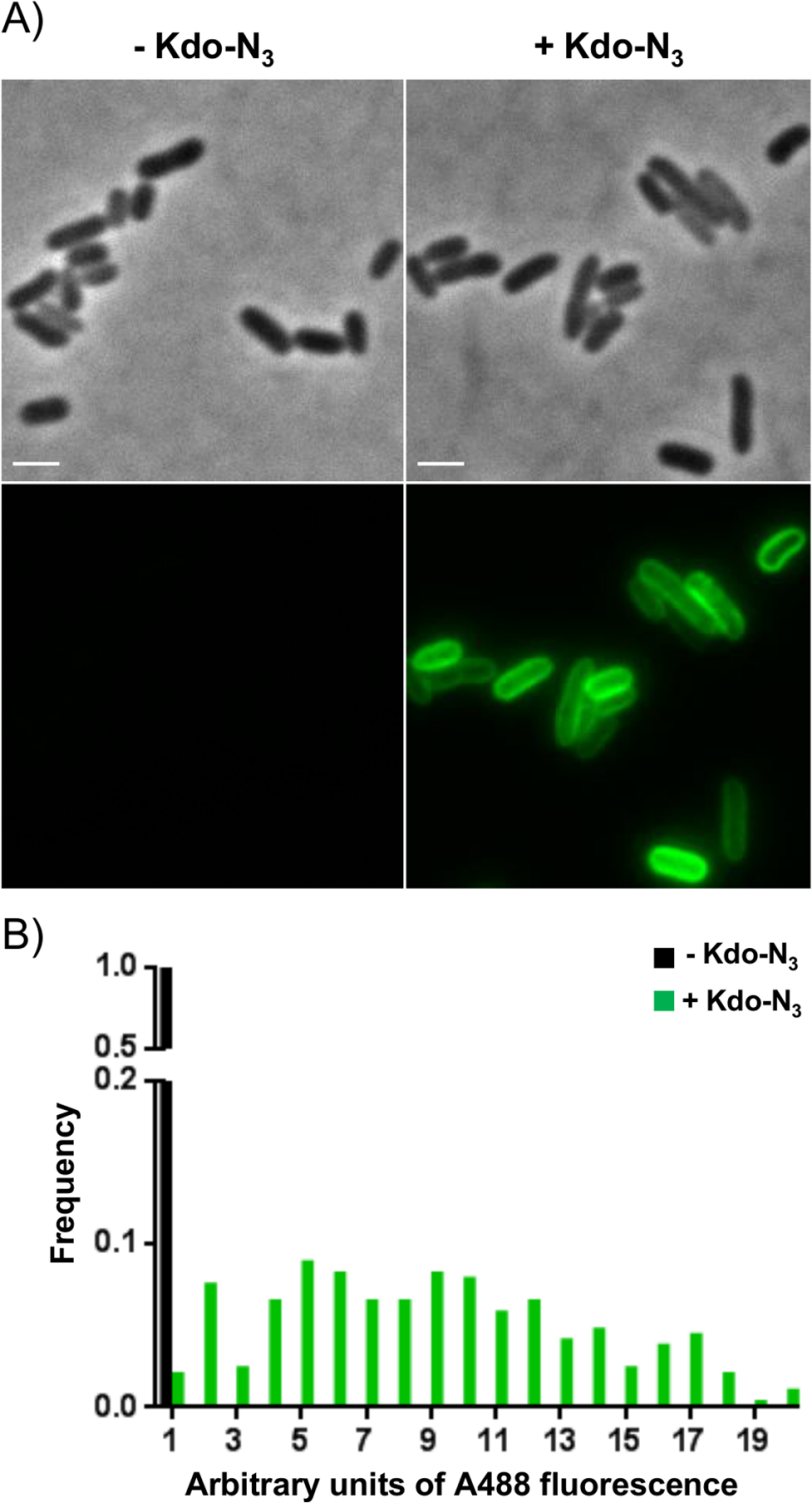
Kdo-N_3_ metabolically labels *E*. *coli* LPS. (A) Metabolically incorporated Kdo-N_**3**_ in *E*. *coli* was revealed by copper-free click chemistry (sulfo-DBCO-biotin followed by an anti-biotin A488 antibody). (B) Frequency distribution of the bacterial fluorescence values in the presence (green bars) or absence of Kdo-N_**3**_ (black bars). Scale bar = 1 μm. More than ten independent experiments have been performed and a representative experiment is depicted.

We have previously demonstrated that 8-azido-3,8-dideoxy-d-manno-octulosonate (Kdo-N_3_) can be metabolically assimilated and incorporated into *E*. *coli* lipopolysaccharides (LPS) and further detected by fluorescent labeling using Copper-Catalyzed Azide-Alkyne Cycloaddition (CuAAC) [22] (Fig 1). Because the conditions used in copper-catalyzed click chemistry are lethal to *E*. *coli*, we wondered whether copper-free click chemistry using a strained cyclooctyne conjugate [21] could be used as a non-lethal alternative, which potentially would not disturb bacterial growth (Fig 1). For this purpose, we monitored Kdo-N_3_ incorporation by culturable *E*. *coli* through reaction with a cyclooctyne-biotin conjugate, followed by anti-biotin-A488 antibody staining. A sulfo-azadibenzocyclooctyne-biotin conjugate (named sulfo-DBCO-biotin in the document) was selected as a compromise for good stability and speed of reaction due to the azadibenzocyclooctyne moiety [28, 29], as well as aqueous solubility and reduced unspecific labeling thanks to the sulfo-β-alanine spacer [30]. Microscopic analyses indicated that Kdo-N_3_ could efficiently be detected at the level of *E*. *coli* outer membrane (Fig 2A) by copper-free click chemistry. The absence of *E*. *coli* growth inhibition due to sulfo-DBCO-biotin was checked by CFU counts (S2 Fig). In addition, as depicted in Fig 2B, fluorescence distribution frequencies indicated that more than 98% of the total *E*. *coli* cells were labeled, which is in agreement with the percentage of bacteria that formed colonies (S2 Fig).

These results demonstrate that copper-free click chemistry, with an adapted cyclooctyne conjugate, allows the detection of culturable Kdo-N_3_-incorporating *E*. *coli* without affecting bacterial growth.

### Specific detection of culturable *E*. *coli* in the presence of dead *E*. *coli*

In a second step, we wondered whether it was possible to differentiate and detect culturable *E*. *coli* within a bacterial mixture composed of dead and culturable *E*. *coli* (1:1 ratio). To easily differentiate these two bacterial populations, we mixed *E*. *coli* with dead (autoclaved) m-cherry *E*. *coli*, both cultured in the presence of Kdo-N_3_ and analyzed the detection of culturable bacteria through sulfo-DBCO-biotin click ligation followed by anti-biotin A488 antibody labeling. Preliminary experiments were undertaken on each bacterial population to determine thresholds of m-cherry fluorescence (m-cherry negative cells = culturable *E*. *coli*, m-cherry positive cells = dead *E*. *coli*) and A488 fluorescence (A488 negative cells = absence of Kdo-N_3_ incorporation, A488 positive cells = Kdo-N_3_ incorporation) (S1 File.).

**Fig 3:**
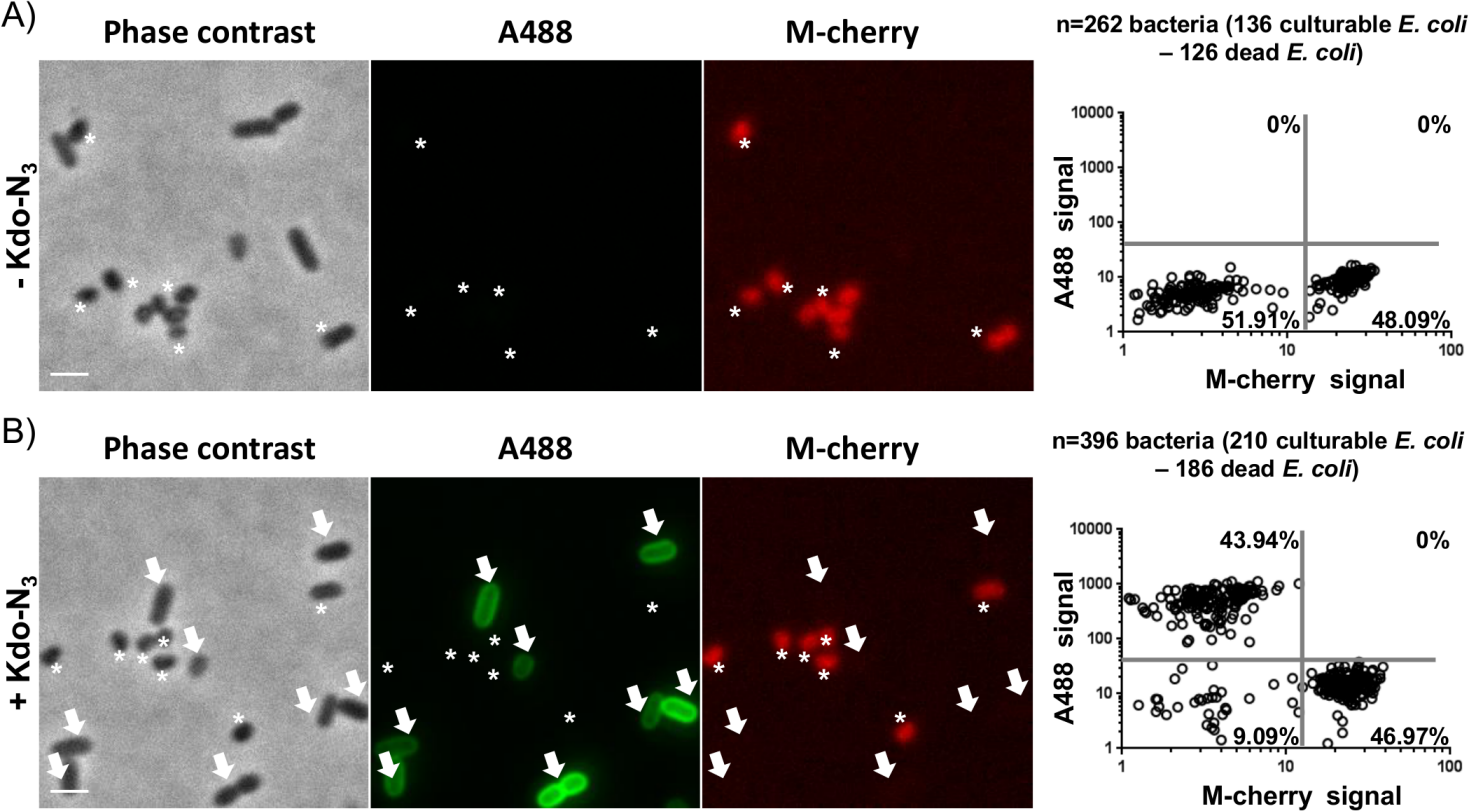
Kdo-N_3_ specifically labels culturable *E*. *coli*. Detection of culturable *E*. *coli* (arrows) mixed with m-cherry dead *E*. *coli* (asterisk) (ratio = 10^6^:10^6^) by copper-free click chemistry (sulfo-DBCO-biotin followed by an anti-biotin A488 antibody) in the absence (A) or presence (B) of metabolically incorporated Kdo-N_**3**_. Scale bar = 1 μm. Frequency distribution of each bacterial fluorescence values in the absence (A) or presence of Kdo-N_**3**_ (B). Four independent experiments have been performed and a representative experiment is depicted.

When we applied these two fluorescence thresholds on the bacterial mixture, we distinguished four compartments which defined four subpopulations: m-cherry^-^/A488^+^ = culturable *E*. *coli*, m-cherry^+^/A488^-^ = dead *E*. *coli*, m-cherry^-^/A488^-^ = false negatives and m-cherry^+^/A488^+^ = dead *E*. *coli* inappropriately detected as culturable *E*. *coli*. Therefore, we were able to deduce percentages of each sub-population within the *E*. *coli* mixture and compare them with initial CFU counts. As expected, in the absence of Kdo-N_3_, two distinct populations were detected on the basis of m-cherry fluorescence threshold: 52% of culturable *E*. *coli* (m-cherry^-^) and 48% of dead *E*. *coli* (m-cherry^+^) (Fig 3A). In the presence of Kdo-N_3_, we detected two major populations in equal proportion: 44% of culturable *E*. *coli* (m-cherry^-^/A488^+^) and 47% of dead *E*. *coli* (m-cherry^+^/A488^-^) (Fig 3B) together with 9% of double negative cells for m-cherry and A488 correspond to both m-cherry dead *E*. *coli* which lost m-cherry fluorescence after the autoclaving process (Figures C and D in S1 File.) and to the small fraction of culturable *E*. *coli* which are not detected by click chemistry (Fig 2B). Our approach did not label any dead *E*. *coli* (0% of m-cherry^+^/A488^+^ cells).

Altogether, these results indicate that our method can differentiate culturable *E*. *coli* from dead *E*. *coli* with good specificity.

### Specific detection of culturable *E*. *coli* (Gram negative) in the presence of *B*. *subtilis* (a model, non Kdo-containing organism)

We have previously shown that *B*. *subtilis* was not detected through copper-catalyzed click labeling after incubation in the presence of Kdo-N_3_ which strongly indicated that Kdo-N_3_ was not assimilated [22]. However, no experiments have yet been undertaken on bacterial mixtures composed of culturable *B*. *subtilis* and *E*. *coli* to assess whether differentiation was possible in such a situation. To address this question, we examined the detection, through sulfo-DBCO-biotin ligation followed by anti-biotin A594 antibody labeling, of *E*. *coli* in a mixture composed of culturable Kan^r^ *E*. *coli* and GFP Chl^r^ *B*. *subtilis* (ratio 1:3). Similarly to the study described above with culturable and dead *E*. *coli*, we first determined thresholds of GFP fluorescence (GFP positive cells = *B*. *subtilis*, GFP negative cells = *E*. *coli*) and A594 fluorescence (A594 negative cells = absence of Kdo-N_3_ incorporation, A594 positive cells = Kdo-N_3_ incorporation) (S2 File).

**Fig 4:**
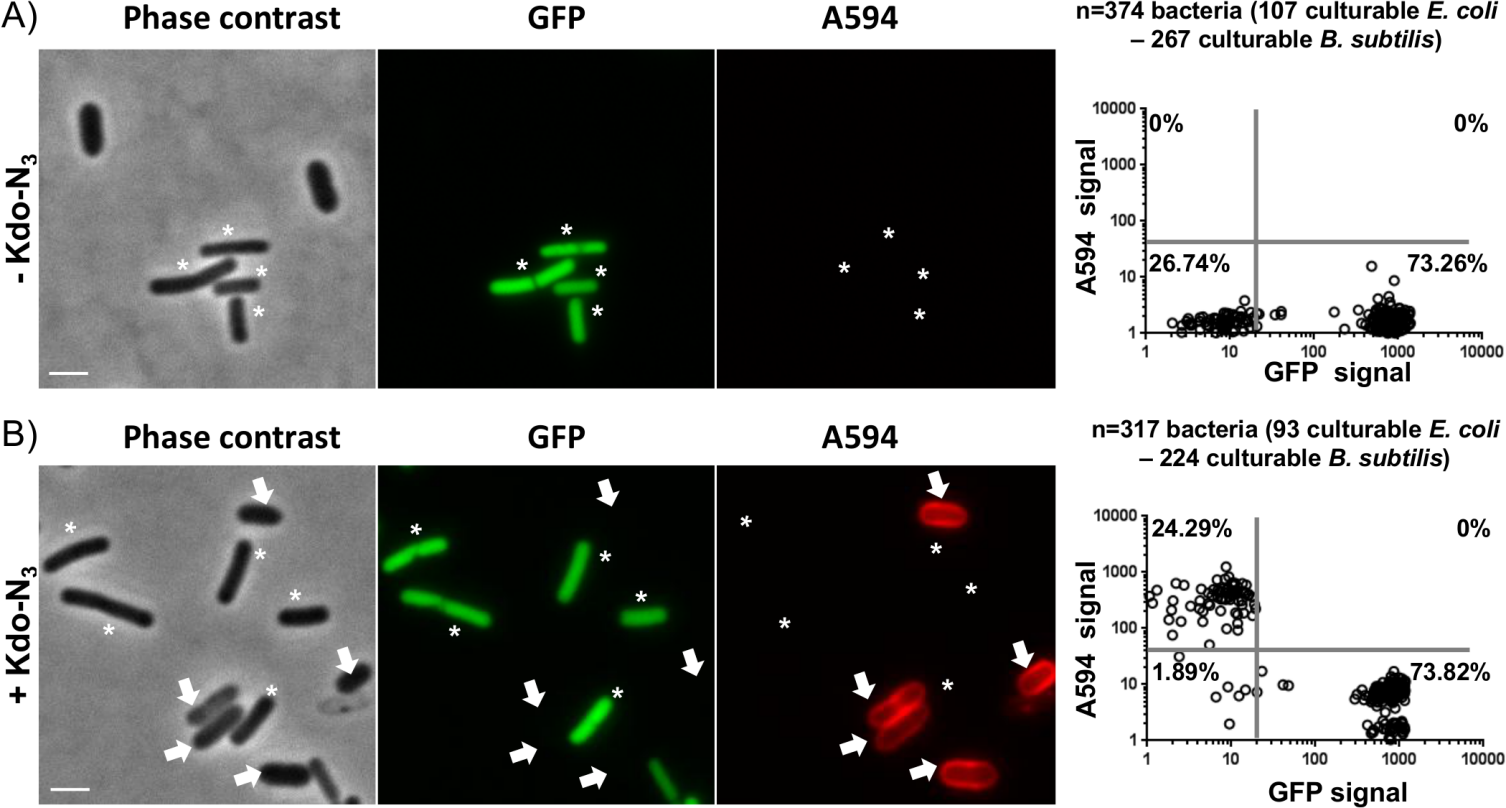
Kdo-N_3_ specifically labels *E*. *coli* culturable Gram negative bacterial membrane. Detection of culturable *E*. *coli* (arrows) mixed with culturable GFP *B*. *subtilis* (asterisk) (ratio = 10^6^:3x10^6^) by copper-free click chemistry (sulfo-DBCO-biotin followed by an anti-biotin A594 antibody) in the absence (A) or presence (B) of metabolically incorporated Kdo-N_**3**_. Scale bar = 1 μm. Frequency distribution of each bacterial fluorescence values in the absence (A) or presence of Kdo-N_**3**_ (B). Five independent experiments have been performed and a representative experiment is depicted.

When we applied these two fluorescence thresholds on the bacterial mixture, we were able to distinguish two main populations. When cultured in the absence of Kdo-N_3_, 26.7% of *E*. *coli* (GFP^-^) and 73.3% of *B*. *subtilis* (GFP^+^) were identified on the basis of GFP fluorescence threshold (Fig 4A). In the presence of Kdo-N_3_, 24.3% of *E*. *coli* (GFP^-^/ A594^+^) and 73.8% of *B*. *subtilis* (GFP^+^/ A594^-^) were detected (Fig 4B). We obtained a negligible subpopulation containing double negative cells for GFP and A594 (1.9%) which corresponded both to *B*. *subtilis* which lost GFP fluorescence (Figures A and B in S2 File) and to the small fraction of culturable *E*. *coli* not labeled after click chemistry (Fig 2B). We did not detect *B*. *subtilis* bacteria falsely labeled as culturable *E*. *coli* (0% of GFP^+^/ A594^+^).

Therefore, these results clearly demonstrate that the method can differentiate culturable Gram negative *E*. *coli* (Kdo^+^) from *B*. *subtilis* (Kdo^-^) with good specificity.

### Specific enrichment and concentration of culturable *E*. *coli* from *B*. *subtilis* using magnetic beads and azide-cyclooctyne ligation

In this work, we have demonstrated that our approach is able to detect culturable *E*. *coli* within a bacterial mixture with a good specificity. We then designed a method to enrich these clicked and culturable cells of interest from a sample, using magnetic bead separation.

**Fig 5:**
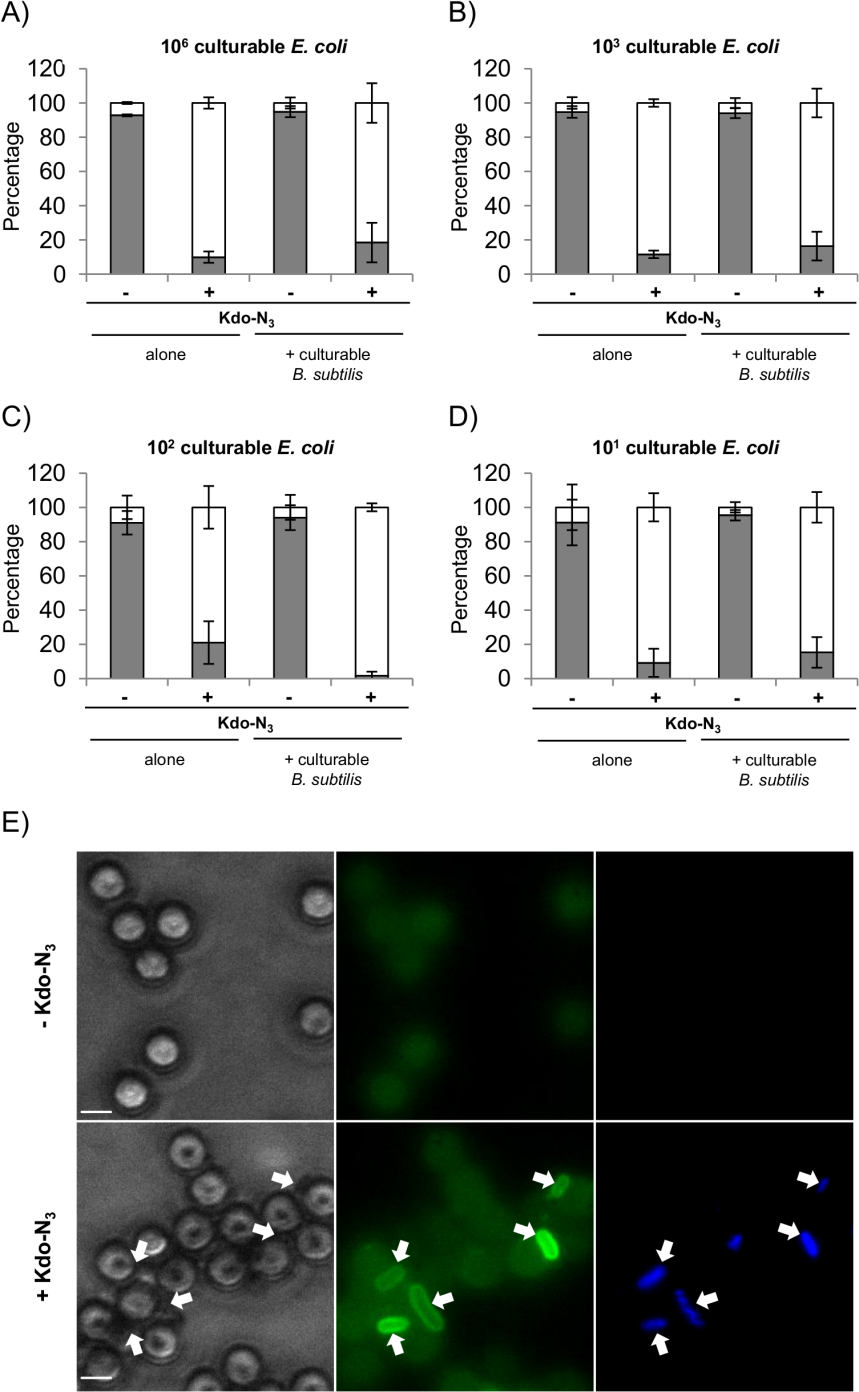
Specific isolation of culturable *E*. *coli* among a bacterial mix with magnetic beads. Determination of culturable *E*. *coli* recovery in the supernatant (grey bars) and magnetic streptavidin bead fraction (white bars) with or without incorporation of Kdo-N_**3**_ followed by copper-free click chemistry (sulfo-DBCO-biotin). Different amount of culturable *E*. *coli* alone or mixed with 10^6^ *B*. *subtilis* were tested: 10^6^ (A), 10^3^ (B), 10^2^ (C), 10^1^ (D). Pictures of culturable *E*. *coli* (arrows) recovered or not in the magnetic streptavidin bead fraction either in the presence or absence of Kdo-N_**3**_, respectively (E). Bacteria were stained with DAPI and Kdo-N_**3**_ incorporation within LPS was revealed with an anti-biotin A488 antibody. Scale bar = 1 μm. Data are means ± SD of four independent experiments.

For this purpose, we first tested the isolation of 10^6^ culturable *E*. *coli* per ml of buffer using magnetic beads (ratio 1 (*E*. *coli*):20 (beads)). When cultured without Kdo-N_3_, the majority of *E*. *coli* (92.8 +/- 0.6%) remained in the supernatant, whereas *E*. *coli* cultured in the presence of Kdo-N_3_ were mainly found in the bead fraction (90.1 +/- 3.3%) (Fig 5A and 5E, Table 1). Without Kdo-N_3_, around 7.2% of *E*. *coli* cells were retained on the magnetic beads, indicating a low aspecificity of this procedure (Table 1). Therefore, we investigated the efficiency of the retention of bacteria in the bead fraction when reducing the concentration of culturable *E*. *coli* down to 10^1^ per ml of buffer and we were still able to extract almost all culturable *E*. *coli* cells (88.4 +/- 2.2%; 79.0 +/- 12.4%; 90.9 +/- 8.2% for 10^3^, 10^2^ and 10^1^ *E*. *coli*, respectively) (Fig 5B–5D and Table 1). As judged by our experimental procedure, recovery efficiency is not affected by *E*. *coli* cell concentration (range 10^6^ to 10^1^ CFU ml^-1^) (p-value>0.05). Therefore we also evaluated whether unwanted bacteria composing the mixture were present in the bead fraction. Interestingly, using the magnetic bead isolation technique, *B*. *subtilis* cells were very poorly collected (a mean of 0.4%) with or without culturable *E*. *coli* for any bacterial ratio tested (S3 Fig and S1 Table). The specific retention in the bead fraction of each bacterium used in this study is summarized in Tables 1 and 2. As depicted in Table 1 and Fig 5, independently of the *E*. *coli* concentration initially used (from 10^1^ to 10^6^ *E*. *coli* ml^-1^), similar proportions of *E*. *coli* cells were isolated by the beads in the presence or absence of 10^6^ *B*. *subtilis* (p-value>0.05). These results demonstrate that *E*. *coli* can be successfully collected with magnetic beads without significant interference of *B*. *subtilis*. For instance, starting with a 1:1000 ratio of *E*. *coli* versus *B*. *subtilis*, we obtained a ratio of 1:3.1 after magnetic beads separation. Finally, starting from an initial working volume of 1 ml, the magnetic beads could be resuspended in a minimum volume of 10 μl leading to a 100 fold concentration of the sample.

**Table 1:** Percentage of culturable *E*. *coli* recovery mixed or not with culturable *B*. *subtilis* in the supernatant fraction and magnetic streptavidin beads fraction with or without incorporation of Kdo-N_3_.

		Supernatant		Magnetic beads	
		- Kdo-N_3_	+ Kdo-N_3_	- Kdo-N_3_	+ Kdo-N_3_
**10** ^**6**^ ***E*. *coli***	**Alone**	92.8 (+/- 0.6)	9.9 (+/- 3.3)	7.2 (+/- 0.6)	90.1 (+/- 3.3)
	**+ 10** ^**6**^ ***B*. *subtilis***	94.9 (+/- 3.2)	18.4 (+/- 11.6)	5.1 (+/- 3.2)	81.6 (+/- 11.6)
**10** ^**3**^ ***E*. *coli***	**Alone**	94.8 (+/- 3.4)	11.6 (+/- 2.2)	5.3 (+/- 3.4)	88.4 (+/- 2.2)
	**+ 10** ^**6**^ ***B*. *subtilis***	94.0 (+/- 2.9)	16.4 (+/- 8.4)	6.0 (+/-2.9)	83.6 (+/- 8.4)
**10** ^**2**^ ***E*. *coli***	**Alone**	91.0 (+/- 6.9)	21.0 (+/- 12.4)	9.0 (+/- 6.9)	79.0 (+/- 12.4)
	**+ 10** ^**6**^ ***B*. *subtilis***	93.9 (+/- 7.3)	1.6 (+/- 2.3)	6.1 (+/- 7.3)	98.4 (+/- 2.3)
**10** ^**1**^ ***E*. *coli***	**Alone**	91.1 (+/- 13.3)	9.1 (+/- 8.2)	8.9 (+/- 13.3)	90.9 (+/- 8.2)
	**+ 10** ^**6**^ ***B*. *subtilis***	95.4 (+/- 3.0)	15.2 (+/- 8.9)	4.6 (+/- 3.0)	84.8 (+/- 8.9)

mean in percentage (+/- standard deviation)

**Table 2:** Percentage of culturable bacteria recovery in the supernatant fraction and magnetic streptavidin beads fraction with or without incorporation of Kdo-N_3_.

	Supernatant		Magnetic beads	
	- Kdo-N_3_	+ Kdo-N_3_	- Kdo-N_3_	+ Kdo-N_3_
***E*. *coli***	94.0	13.4	6.1	86.6
***B*. *subtilis***	99.9	99.6	0.1	0.4

mean in percentage

Altogether these results indicate that our technology permits the specific recovery of the large majority of culturable Gram negative *E*. *coli* from *B*. *subtilis* as well as their concentration.

### Minimal Kdo-N_3_ assimilation time allowing specific *E*. *coli* enrichment

**Fig 6:**
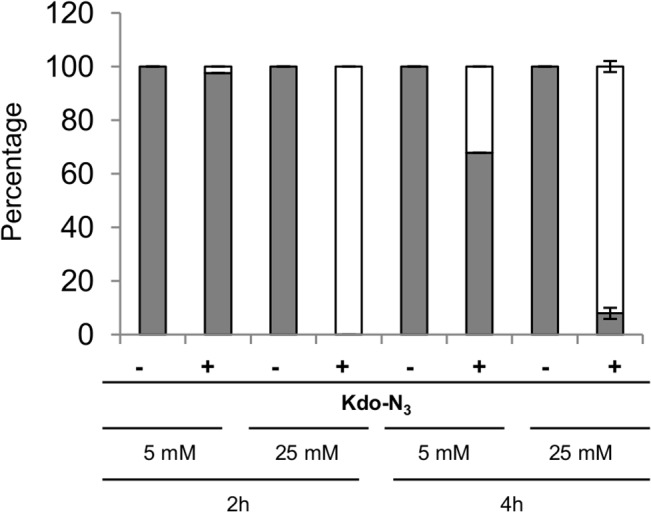
Isolation of all culturable *E*. *coli* cells incubated with 25 mM of Kdo-N_3_ within 2 h. Determination of culturable *E*. *coli* recovery in the supernatant (grey bars) and magnetic streptavidin bead fraction (white bars) with or without incorporation of Kdo-N_**3**_ 5mM or 25 mM for 2 and 4 h followed by copper-free click chemistry (sulfo-DBCO-biotin). Data are means ± SD of four independent experiments.

In all previous experiments, *E*. *coli* were cultured overnight in the presence of Kdo-N_3_. We finally investigated the minimal period of Kdo-N_3_ assimilation allowing retention of the majority of *E*. *coli* cells onto magnetic beads after azide-cyclooctyne ligation. For this purpose, approximately 5 culturable *E*. *coli* per ml of medium were incubated for 2 to 4 hours in the presence of various concentrations of Kdo-N_3_ (0 to 25 mM, 25 mM being the maximal Kdo concentration allowing normal *E*. *coli* cell growth as indicated in S4 Fig) and *E*. *coli* cells trapped by magnetic beads were eventually quantified. Analyses depicted in Fig 6 demonstrates that 2 h of 25 mM Kdo-N_3_ incubation was sufficient to isolate the majority of culturable *E*. *coli* cells present in a sample.

In conclusion, these data highlight an efficient application of our technique to rapidly and specifically enrich and concentrate *E*. *coli* culturable Gram negative bacteria of interest in the presence of *B*. *subtilis*.

## Discussion

The innovative technology described above, which is based on the combination of cell tagging through non-lethal click chemistry and sorting with magnetic beads, is dedicated to rapidly detect, enrich and concentrate culturable *E*. *coli* from *B*. *subtilis* present in an initial sample, both used as model organisms containing or not Kdo, respectively.

The technique explored in this study appears to be highly attractive and interesting for many reasons. In our previous study, that demonstrated metabolic assimilation and incorporation of Kdo-N_3_ into *E*. *coli* LPS_,_ Copper-Catalyzed Azide-Alkyne Cycloaddition (CuAAC) has been used, leading to bacterial lethality as a side effect [22]. Here, in order to avoid this toxic effect, we explored copper-free Strain-Promoted Alkyne-Azide Cycloaddition (SPAAC) (Fig 1). Indeed, it has been observed that this copper-free version of click chemistry was not toxic to eukaryotic cells [21]. We report that this alternative click chemistry is also non-lethal to bacteria such as *E*. *coli*. Moreover, this procedure is as efficient as copper-catalyzed click chemistry since the time of reaction required is identical (30 min) and the use of the same final concentration of alkyne provides exactly the same labeling efficiency, since 98% of *E*. *coli* are labeled.

Thanks to non lethal copper-free azide-cyclooctyne click chemistry we further confirmed the hypothesis formulated by Dumont *et al*., [22] that only culturable Gram negative bacteria (*E*. *coli*) were detected after assimilation and membrane incorporation of Kdo-N_3_ (active LPS biosynthetic and transport pathway). Indeed, we show that dead Gram negative *E*. *coli* or living Gram positive *B*. *subtilis*, as a model non Kdo-containing organism, are not tagged by our procedure. Moreover, using this variant of click chemistry, we demonstrate the possibility to enrich culturable *E*. *coli*, by specific isolation, present among *B*. *subtilis*, using magnetic beads. Taking into account the results [22] indicating that all Gram negative bacteria tested in the study could be detected *via* the assimilation of Kdo-N_3_ (excepted for the *Shewanella* genus) [25, 26], we can assume that, like *E*. *coli* in this work, these Gram negative bacteria will also be trapped using magnetic beads.

The possibility given by the present technology to isolate and concentrate/enrich culturable Gram negative bacteria from a sample to be analyzed could, for example, reduce the pre-culture time required to obtain the 10^3^ bacteria ml^-1^ (*E*. *coli* O157, *Salmonella*…) necessary for subsequent PCR analysis or ELISA tests in routine assays [31]. For instance, here we have been able to concentrate *E*. *coli* 100 fold (from 1 ml to 10 μl). If we assume that the same procedure could be efficiently applied to all kinds of Gram negative bacteria and to the higher volume usually used for detection of microbial presence in food industry (250 ml), we would be able to concentrate bacteria up to 25 000 fold. Moreover, in the hypothesis where only 1 *E*. *coli* (doubling time 20 min) is present in the 250 ml sample tested and that this cell starts to divide immediately (no latency), 6 h are necessary to get the 10^3^ bacteria.ml^-1^ required for further PCR identification analyses for example. Using our technology, after 2 h of growth in presence of Kdo-N_3_ followed by magnetic bead retention (1 h procedure), this same bacterium concentration is obtained. Consequently, we would be able not only to reduce by a factor of 2 the time of analysis but also to isolate specifically culturable Gram negative bacteria from a sample in the meanwhile.

In various diagnostic areas like environmental, biological or food processing controls, it is necessary to determine the potential presence of a set of bacteria of interest. One can easily imagine the importance to isolate at the same time a wide variety of culturable bacteria present in a complex matrix in order to further identify them and determine the quantity of each of them. Interestingly, another remarkable advantage provided by our technology would be to permit, in one shot, the concentration and the enrichment of only all culturable Gram negative bacteria among other organisms even if they are under represented, in a sample devoid from algae or higher plants cells, which have been shown to possess Kdo within their cell wall potentially leading to interference with our procedure. This aspect still needs to be investigated. Collected culturable Kdo containing organisms like Gram negative bacteria can thereafter be directly used for further identification and enumeration by ELISA or QtPCR since our technology allows to concentrate bacteria in a smaller volume (10 μl in this study). More interestingly, collected bacteria can also be analyzed through bacterial culture on plate since our procedure does not affect cell viability.

All together, even if the present technology will still have to be optimized using higher culture volumes and then be applied to environmental samples, we are convinced that this new procedure is already highly attractive. Moreover, the possibility to enrich culturable Gram negative bacteria directly from a sample mixture of bacteria opens new horizons by allowing to raise specific questions on this bacterial sub-population.

## Supporting Information

S1 File Determination of fluorescence signal thresholds of culturable *E*. *coli* and m‐cherry dead *E*. *coli* separately. Pictures and fluorescence signal analyses of culturable *E*. *coli* and m‐cherry dead *E*. *coli* in the absence (Figure A) and (Figure C) and presence (Figure B) and (Figure D), respectively of Kdo‐N_3_ followed by a copper‐free click chemistry (sulfo‐DBCO‐biotin + an anti‐biotin A488 antibody). Scale bar = 1μm.
(TIFF)

S2 File Determination of fluorescence signal thresholds of culturable *E*. *coli* and culturable GFP *B*. *subtilis* separately. Pictures and fluorescence signal analyses of culturable GFP *B*. *subtilis* and culturable *E*. *coli* in the absence (Figure A) and (Figure C) and presence (Figure B) and (Figure D), respectively of Kdo‐N_3_ followed by a copper‐free click reaction (sulfo‐DBCO‐biotin + an anti‐biotin A594 antibody). Scale bar = 1 μm.
(TIFF)

S1 Fig Structures of Kdo‐N_3_ and sulfo‐DBCO‐biotin. (TIFF)

S2 Fig Copper‐free click chemistry does not affect bacterial growth. CFUs scoring of culturable *E*. *coli* in the absence or presence of Kdo‐N_3_ before (dark bars) and after (grey bars) click reaction using sulfo‐DBCO‐biotin. Data are means ± SD of four independent experiments.
(TIFF)

S3 Fig Determination of the magnetic beads isolation technique aspecificity. Scoring of culturable *B*. *subtilis* recovery in the supernatant fraction (grey bars) and magnetic streptavidin beads fraction (white bars) with or without incorporation of Kdo‐N3 followed by copper‐free click chemistry (sulfo‐DBCO‐biotin) within different amounts of culturable *E*. *coli*. Data are means ± SD of four independent experiments.
(TIFF)

S4 Fig Growth of culturable *E*. *coli* cells within different concentration of Kdo‐N3 over 6h. CFU scoring of culturable *E*. *coli* in the absence or presence of different concentrations of Kdo‐N3: 0 mM (circle), 10 mM (square), 25 mM (triangle) and 50 mM (star). Data are means of four independent experiments.
(TIFF)

S1 Table Percentage of culturable *B*. *subtilis* recovery mixed with culturable *E*. *coli* in the supernatant fraction and magnetic streptavidin beads fraction with or without incorporation of Kdo-N_3_. (DOCX)
