# Supplementary figures and images for: Rapid and Specific Enrichment of Culturable Gram Negative Bacteria Using Non-Lethal Copper-Free Click Chemistry Coupled with Magnetic Beads Separation
Source: PLoS One. 2015 Jun 10;10(6):e0127700. doi: 10.1371/journal.pone.0127700 (PMC4465638; doi:10.1371/journal.pone.0127700)

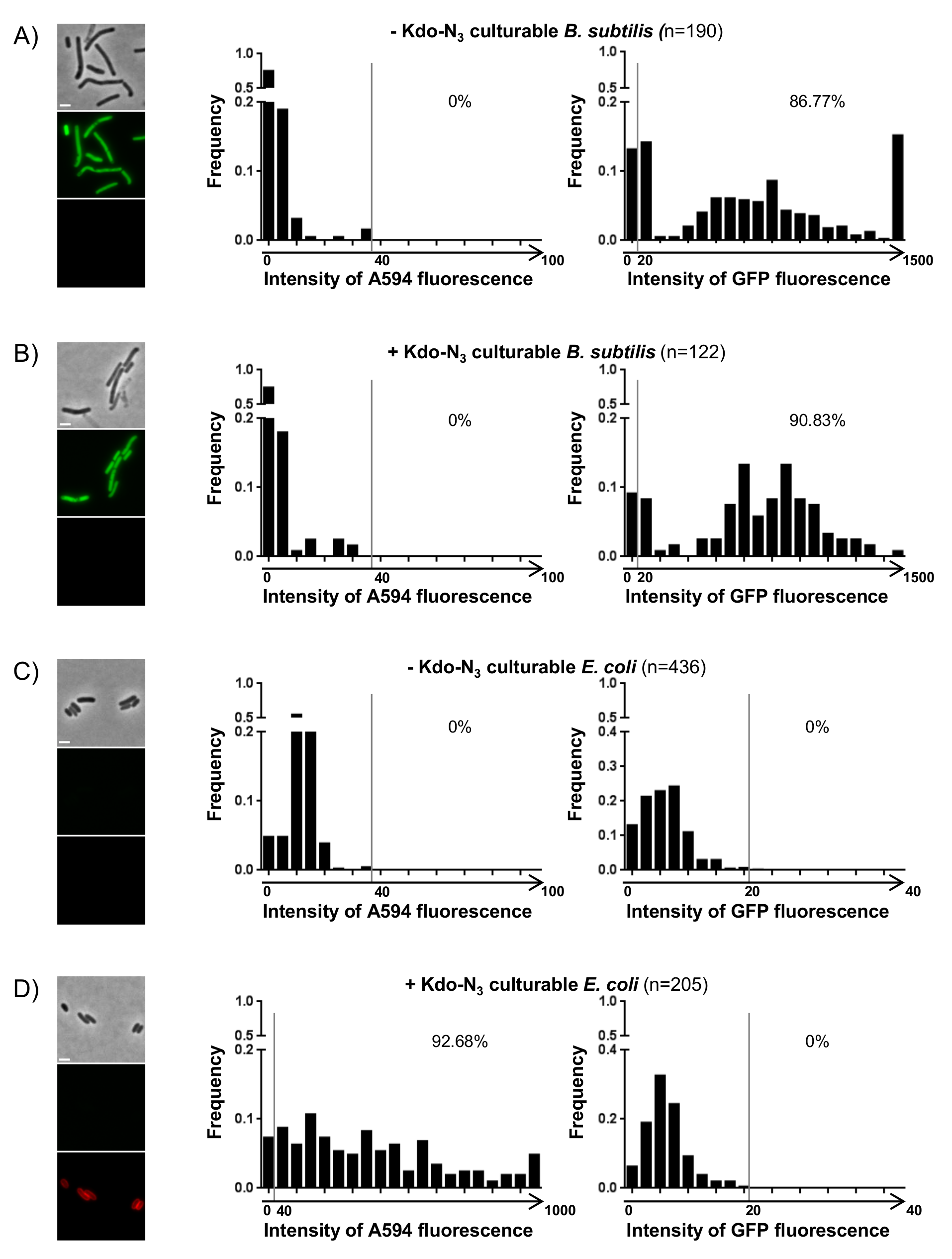

Supplement: S1 File — Pictures and fluorescence signal analyses of culturable E. coli and m‐cherry dead E. coli in the absence (Figure A) and (Figure C) and presence (Figure B) and (Figure D), respectively of Kdo‐N3 followed by a copper‐free click chemistry (sulfo‐DBCO‐biotin + an anti‐biotin A488 antibody). Scale bar = 1μm. (TIFF) [file pone.0127700.s001.TIFF]

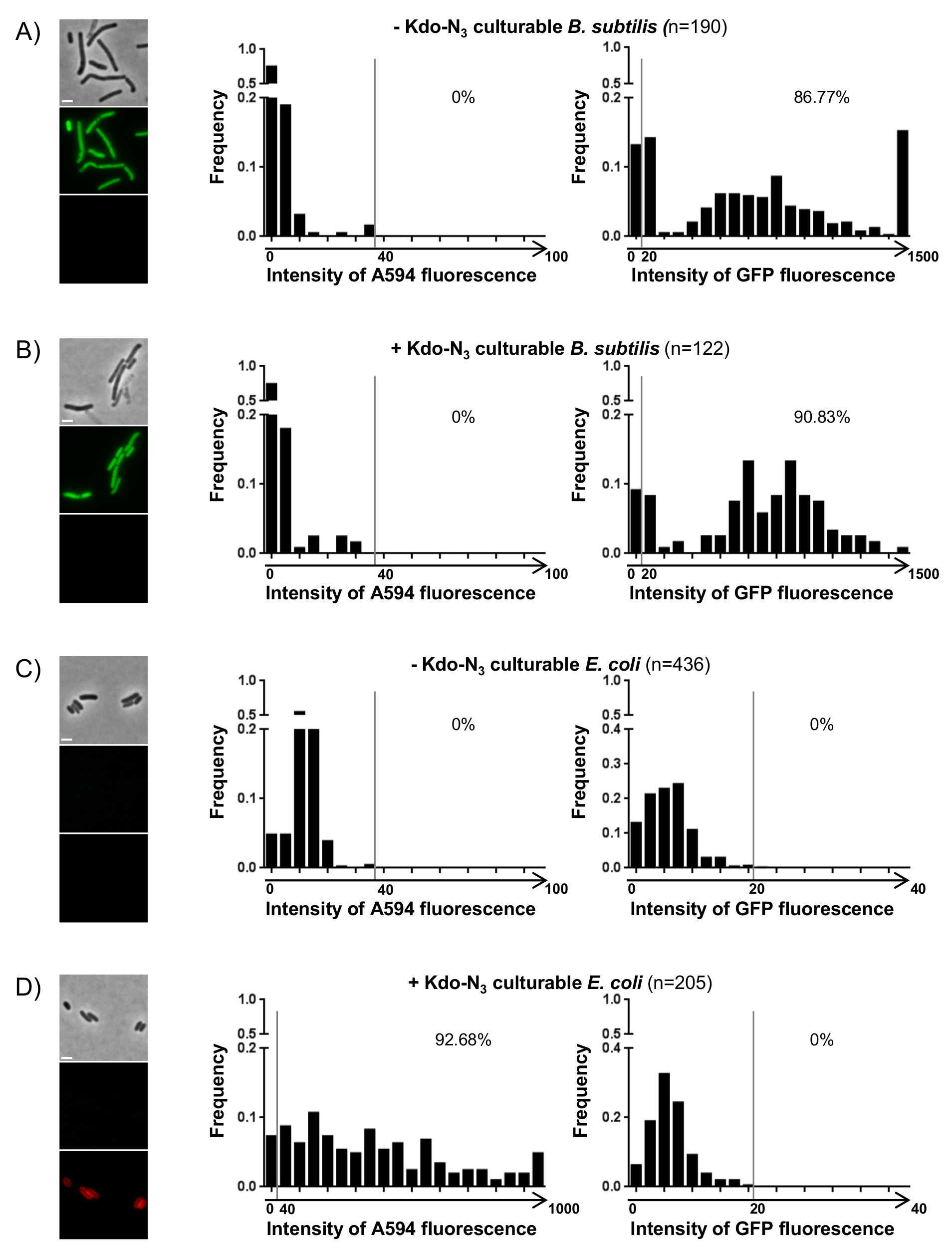

Supplement: S2 File — Pictures and fluorescence signal analyses of culturable GFP B. subtilis and culturable E. coli in the absence (Figure A) and (Figure C) and presence (Figure B) and (Figure D), respectively of Kdo‐N3 followed by a copper‐free click reaction (sulfo‐DBCO‐biotin + an anti‐biotin A594 antibody). Scale bar = 1 μm. (TIFF) [file pone.0127700.s002.TIFF]

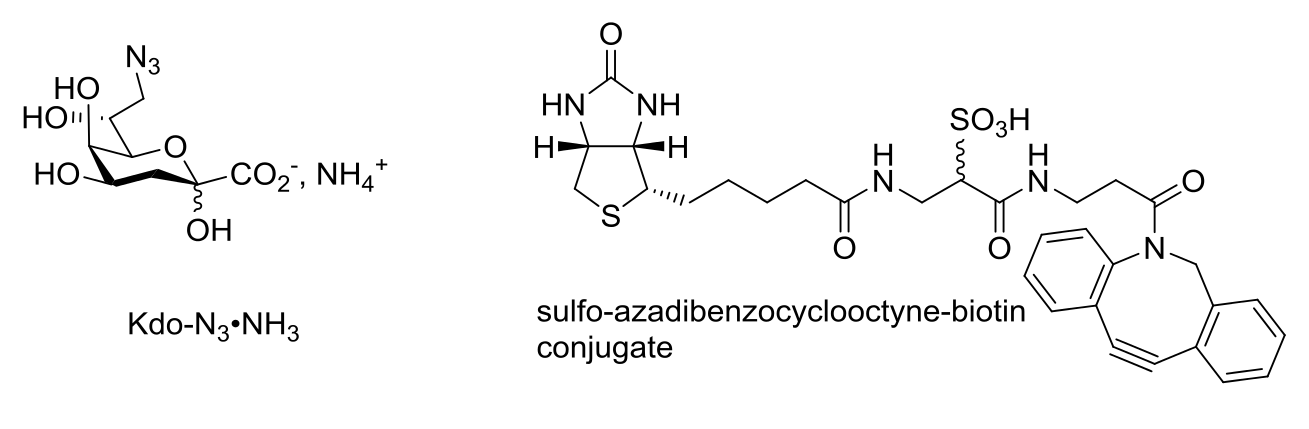

Supplement: S1 Fig — (TIFF) [file pone.0127700.s003.TIFF]

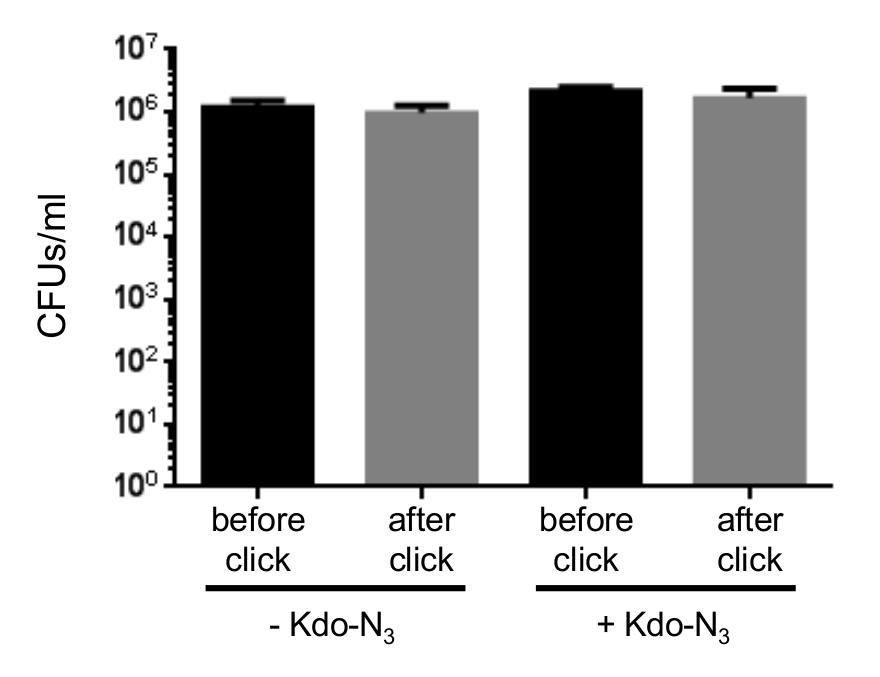

Supplement: S2 Fig — CFUs scoring of culturable E. coli in the absence or presence of Kdo‐N3 before (dark bars) and after (grey bars) click reaction using sulfo‐DBCO‐biotin. Data are means ± SD of four independent experiments. (TIFF) [file pone.0127700.s004.TIFF]

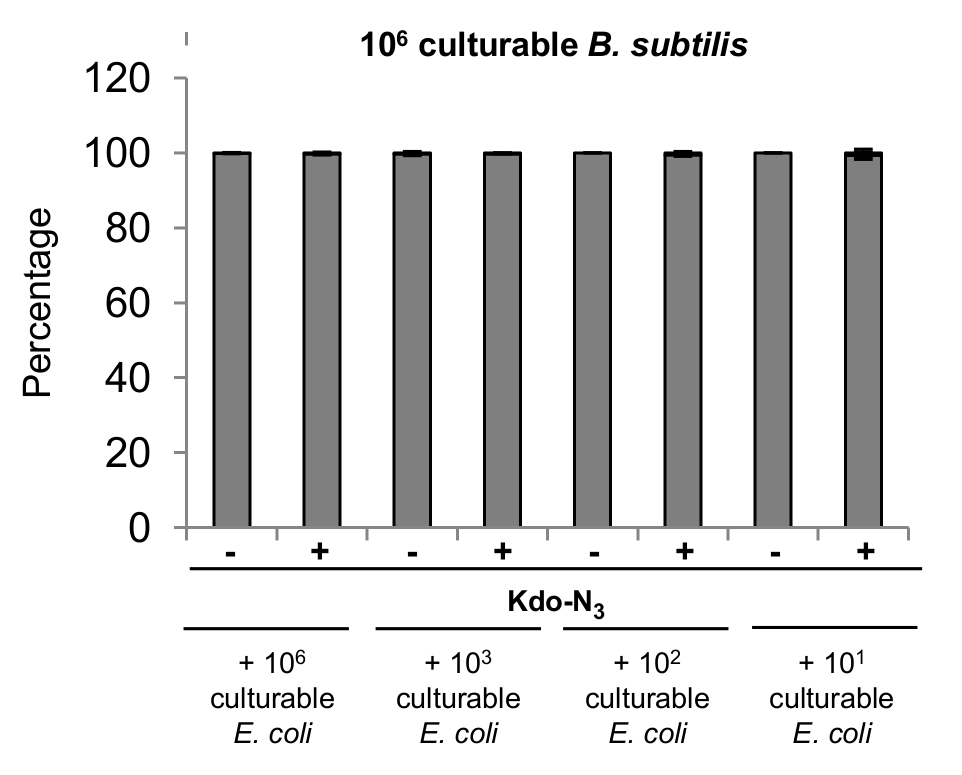

Supplement: S3 Fig — Scoring of culturable B. subtilis recovery in the supernatant fraction (grey bars) and magnetic streptavidin beads fraction (white bars) with or without incorporation of Kdo‐N3 followed by copper‐free click chemistry (sulfo‐DBCO‐biotin) within different amounts of culturable E. coli. Data are means ± SD of four independent experiments. (TIFF) [file pone.0127700.s005.TIFF]

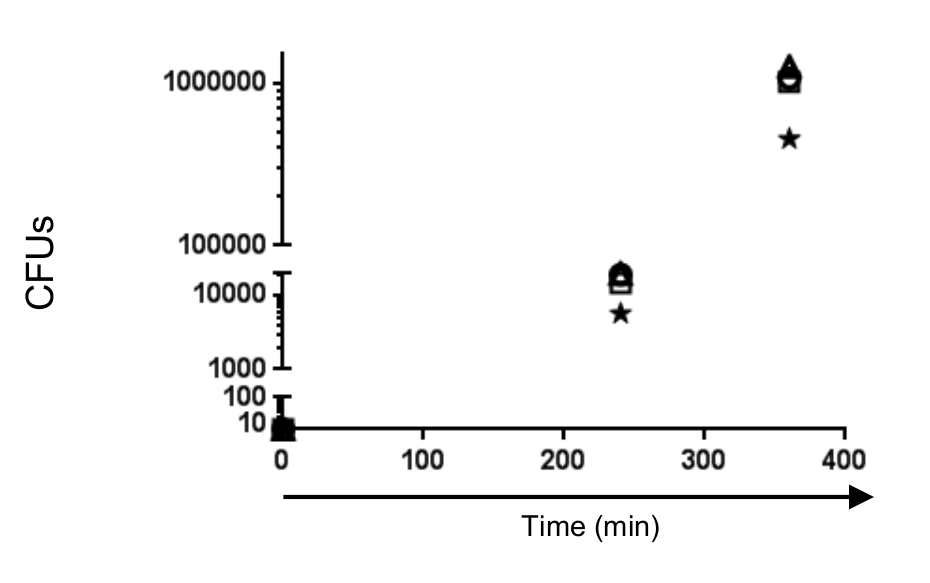

Supplement: S4 Fig — CFU scoring of culturable E. coli in the absence or presence of different concentrations of Kdo‐N3: 0 mM (circle), 10 mM (square), 25 mM (triangle) and 50 mM (star). Data are means of four independent experiments. (TIFF) [file pone.0127700.s006.TIFF]
