# Supplementary material for: Rapid and Specific Enrichment of Culturable Gram Negative Bacteria Using Non-Lethal Copper-Free Click Chemistry Coupled with Magnetic Beads Separation
Source: PLoS One. 2015 Jun 10;10(6):e0127700. doi: 10.1371/journal.pone.0127700 (PMC4465638; doi:10.1371/journal.pone.0127700)
Supplement: S1 Table — (DOCX) [file pone.0127700.s007.docx]

**S1 Table.** Percentage of culturable *B. subtilis* recovery mixed with culturable *E. coli* in the supernatant fraction and magnetic streptavidin beads fraction with or without incorporation of Kdo-N_3_

|  |  | **Supernatant** | | **Magnetic beads** | |
| --- | --- | --- | --- | --- | --- |
|  |  | **- Kdo-N_3_** | **+ Kdo-N_3_** | **- Kdo-N_3_** | **+ Kdo-N_3_** |
| **10^6^ *B. subtilis*** | **+ 10^6^ *E. coli*** | 99.9 (+/- 0.1) | 99.7 (+/- 0.2) | 0.1 (+/- 0.1) | 0.3 (+/- 0.2) |
|  | **+ 10^3^ *E. coli*** | 99.7 (+/- 0.4) | 99.8 (+/- 0.1) | 0.3 (+/- 0.4) | 0.2 (+/- 0.1) |
|  | **+ 10^2^ *E. coli*** | 100.0 (+/- 0.0) | 99.5 (+/- 0.4) | 0.0 (+/- 0.0) | 0.5 (+/- 0.4) |
|  | **+ 10^1^ *E. coli*** | 100.0 (+/- 0.0) | 99.3 (+/- 1.0) | 0.0 (+/- 0.0) | 0.7 (+/- 1.0) |

mean in percentage (+/- standard deviation)
